# Selecting Closely-Linked SNPs Based on Local Epistatic Effects for Haplotype Construction Improves Power of Association Mapping

**DOI:** 10.1534/g3.119.400451

**Published:** 2019-10-11

**Authors:** Fang Liu, Renate H. Schmidt, Jochen C. Reif, Yong Jiang

**Affiliations:** Department of Breeding Research, Leibniz Institute of Plant Genetics and Crop Plant Research (IPK) Gatersleben, 06466 Stadt Seeland, Germany

**Keywords:** haplotype, epistasis, genome-wide association study

## Abstract

Genome-wide association studies (GWAS) have gained central importance for the identification of candidate loci underlying complex traits. Single nucleotide polymorphism (SNP) markers are mostly used as genetic variants for the analysis of genotype-phenotype associations in populations, but closely linked SNPs that are grouped into haplotypes are also exploited. The benefit of haplotype-based GWAS approaches *vs.* SNP-based approaches is still under debate because SNPs in high linkage disequilibrium provide redundant information. To overcome some constraints of the commonly-used haplotype-based GWAS in which only consecutive SNPs are considered for haplotype construction, we propose a new method called functional haplotype-based GWAS (FH GWAS). FH GWAS is featured by combining SNPs into haplotypes based on the additive and epistatic effects among SNPs. Such haplotypes were termed functional haplotypes (FH). As shown by simulation studies, the FH GWAS approach clearly outperformed the SNP-based approach unless the minor allele frequency of the SNPs making up the haplotypes is low and the linkage disequilibrium between them is high. Applying FH GWAS for the trait flowering time in a large *Arabidopsis thaliana* population with whole-genome sequencing data revealed its potential empirically. FH GWAS identified all candidate regions which were detected in SNP-based and two other haplotype-based GWAS approaches. In addition, a novel region on chromosome 4 was solely detected by FH GWAS. Thus both the results of our simulation and empirical studies demonstrate that FH GWAS is a promising method and superior to the SNP-based approach even if almost complete genotype information is available.

Genome-wide association studies (GWAS) have been widely applied to identify candidate regions on chromosomes influencing complex traits in plant (Brachi *et al.* 2011), animal (Goddard and Hayes 2009) and human populations (McCarthy *et al.* 2008). The most commonly used genetic variants to test genotype-phenotype associations in GWAS are single nucleotide polymorphism (SNP) markers. Alternatively, SNPs can be combined into haplotypes which has been popular in association studies since the structure of human haplotype blocks was revealed (Gabriel *et al.* 2002; Cardon and Abecasis 2003). Empirical studies showed that haplotype-based GWAS was able to detect loci which failed to be identified in single SNP-based GWAS (Trégouët *et al.* 2009; Pryce *et al.* 2010). Nonetheless, contrasting results comparing the power of haplotype- and SNP-based GWAS were reported in previous studies (Lorenz *et al.* 2010) and whether it is beneficial to use haplotypes as variants in GWAS has to be evaluated on a case-by-case basis (Long and Langley 1999).

Potential advantages for testing associations between phenotypes and haplotypes, instead of SNP markers include: haplotypes may exploit epistatic interactions among markers within the haplotype blocks (Schaid 2004); contain more information on whether two alleles are identical by decent (Meuwissen and Goddard 2000); utilize the information from evolutionary history (Durrant *et al.* 2004) and provide more power than single SNPs when multiple alleles contribute to the trait (Morris and Kaplan 2002). There are, however, also drawbacks when using haplotypes as variants in association tests. Adding irrelevant markers to a possible causal genetic variant will dilute the contrasts among haplotype allele classes (Clark 2004). A haplotype consisting of *k* SNPs may have up to *2^k^* different haplotype alleles, which will increase the degree of freedom and hence reduce the power of test (Zhao *et al.* 2003).

Among factors affecting the power of haplotype-based GWAS approaches, a fundamental one is how the haplotypes are constructed. The widely used methods group SNPs by sliding-windows of fixed or variable length (Lin *et al.* 2004; Huang *et al.* 2007), by the linkage disequilibrium (LD) between adjacent SNPs (Barrett *et al.* 2005) or by the diversity of haplotypes across samples (Zhang *et al.* 2002; Anderson and Novembre 2003). Common to these methods is that only consecutive SNPs, which are often in high LD, are combined into haplotypes. Consequently, in many cases the haplotypes are not much more informative than a single SNP because the SNPs in high LD provide redundant information (Laramie *et al.* 2007). This may provide one explanation for the contradicting results reported in the literature comparing the power of haplotype- and SNP-based GWAS approaches. Other methods were developed to search for haplotypes consisting of most informative and possibly non-consecutive SNPs within a certain region (Laramie *et al.* 2007; Yu and Schaid 2007; Abo *et al.* 2008; Yang *et al.* 2008; Dai *et al.* 2009; Knuppel *et al.* 2012). Despite their potential, the high computational burden associated with these methods restricted their use mainly to association studies for candidate gene regions.

In this study we addressed these limitations by developing a new method of constructing haplotypes, taking epistatic effects among SNPs into account. Epistasis has been identified as an important contributor to the genetic variation of complex quantitative traits (Carlborg and Haley 2004; Mackay 2014). It has been reported for two- or three-locus examples that a model involving haplotype effects can be reparametrized into one including the main and epistatic effects among markers constituting the haplotypes (Conti and Gauderman 2004; Schaid 2004). More recently this relationship between haplotype and marker effects was formally proved in the framework of genome-wide prediction for homozygous populations (Jiang *et al.* 2018). Capitalizing on these theoretical findings, we exploited epistatic effects among markers for constructing haplotypes and implemented this novel strategy in haplotype-based GWAS for a large *Arabidopsis thaliana* population generated by the 1001 Genomes Consortium (1001 Genomes Consortium 2016). The results were compared to those obtained with two commonly used haplotype-based GWAS methods as well as the single SNP-based approach, and underlined the ability to detect hidden marker-trait associations using the newly devised strategy. Moreover, simulation studies revealed factors which determine whether the developed GWAS approach outperforms the single SNP-based method.

## Materials and Methods

Throughout the manuscript, a combination of (possibly non-consecutive) SNPs was termed haplotype. By haplotype effect we meant to consider the effects of all possible alleles together. When referring to a specific allele, the term haplotype allele was used.

### The baseline model for genome-wide association mapping

A standard linear mixed model controlling the structure of genetic relatedness or the polygenic background effects (Yu *et al.* 2006) was used for genome-wide association mapping. In this study the model was used for testing single SNP effects, epistatic effects among several SNPs and haplotype effects. It can be uniformly described as following:

$$y=1_{n}\mu +X\beta +g+e$$ (1)

where y is a n-dimensional vector of observed phenotypic values (n is the number of genotypes), $1_{n}$ is a vector of one’s, μ is a common intercept term, β represents the effects of the variables (SNPs, interactions among SNPs or haplotype alleles) being tested, X stands for the corresponding design matrix, g is the n-dimensional vector of genotypic effects and e is the residual term. In the model we assume that μ and β are fixed effects, g and e are random effects and $g∼N(0,\sigma_{g}^{2}K)$, $e∼N(0,\sigma_{e}^{2}I)$, where K is a marker-derived kinship matrix, I is the identity matrix, $\sigma_{\text{g}}^{2}$ and $\sigma_{\text{e}}^{2}$ are the corresponding variance components. Distance matrix was calculated with Rogers’ distance (Reif *et al.* 2005) and K was equaling one minus distance matrix. To reduce the computational load, an acceleration algorithm was implemented in which the linear mixed model was transferred to a simple linear model by applying eigen-decomposition to the kinship matrix (Lippert *et al.* 2011). The significance of β was assessed by *t*-test.

### The general procedure of functional haplotype-based GWAS (FH GWAS)

**Figure 1:**
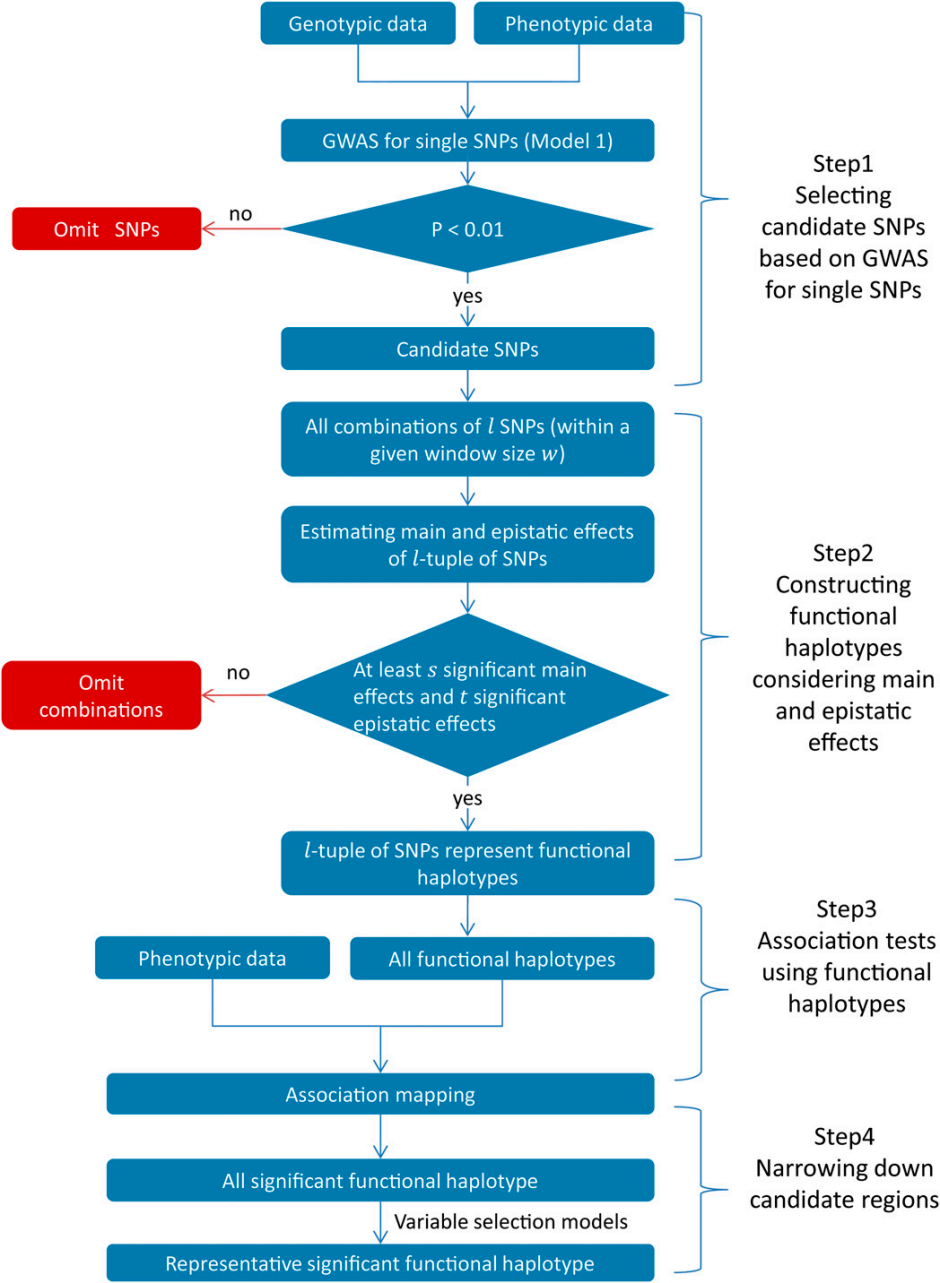
The workflow for FH GWAS.

In genomic prediction, it was demonstrated that modeling haplotype effects is equivalent to modeling main and epistatic effects among markers within the haplotype block, except that the two models assume different covariance structures for the unknown parameters (Jiang *et al.* 2018). The theory also applies to GWAS and in this case the two models are strictly equivalent because the parameters to be tested are assumed to be fixed effects (Equation 1) and hence without any covariance structure. Based on this theory, we developed a new haplotype-based GWAS approach, FH GWAS, with haplotypes based on the main and epistatic effects among SNPs. FH GWAS consists of the following four steps summarized in Figure 1.

#### Step 1: Preselecting SNPs to be combined into haplotypes:

GWAS for single SNPs is performed using the linear mixed model (Equation 1) and a mild threshold without correction for multiple testing is applied to identify candidate SNPs (*e.g.*, *P* < 0.01). SNPs whose P values do not pass the threshold are excluded in subsequent analyses.

#### Step 2: Constructing functional haplotypes:

In this step, candidate SNPs showing significant local epistatic effects are grouped into haplotypes. First we need to determine two parameters: the window size for searching haplotypes (denoted by w) and the number of SNPs in each haplotype (denoted by l). Theoretically the choice of these two parameters can be arbitrary. But in practice one needs to consider the linkage disequilibrium in the population, the computational load and the power of the association test (More details were discussed in the Discussion section). Once the parameters are chosen, GWAS model (Equation 1) is then performed for any l-tuple of SNPs within the window size w, including the additive effects of each SNP and the digenic epistatic effects for each pair of SNPs. That is, the entries in the vector β include $a_{i}$ (1≤i≤l) and $aa_{ij}$ (1≤i<j≤l), where $a_{i}$ denotes the additive effect of the i-th SNP, $aa_{ij}$ denotes the epistatic effects between the i-th and the j-th SNP. In the case that  l  is small, higher-order epistatic effects can also be included in the model. Next we determine the number of significant additive and epistatic effects (again under a mild threshold) required for grouping the l-tuple of SNPs into a haplotype, *i.e.*, when there are at least s (s≤l) significant additive effects and t (t≤l(l−1)/2) significant epistatic effects, the l-tuple of SNPs is combined into a haplotype. Since main as well as epistatic effects are taken into account for haplotype construction we coined the term functional haplotypes. Importantly, for each trait to be analyzed in a given population, a different set of candidate SNPs and functional haplotypes will be obtained.

#### Step 3: GWAS using functional haplotypes:

All resulting functional haplotypes are applied in conjunction with phenotypic data for GWAS using the linear mixed model (Equation 1). Significant functional haplotypes are then identified using a stringent genome-wide threshold corrected for multiple testing, *e.g.*, *P* < 0.05 after Bonferroni (Dunn 1961) or Benjamini-Hochberg correction (Benjamini and Hochberg 1995).

#### Step 4: Narrowing Down candidate regions:

In each region in which significant functional haplotypes are detected in Step 3, we fitted all significant functional haplotypes in a variable-selection model (*e.g.*, the stepwise linear regression model (Draper and Smith 2014) or the least absolute shrinkage and selection operator (LASSO, Tibshirani 1996) to select representative significant functional haplotypes. In any region where significant functional haplotypes are found, the span of all representative haplotypes is considered as a final candidate region.

### Two other methods constructing haplotypes

To compare FH GWAS with existing haplotype-based GWAS approaches, we considered the following two commonly-used methods of constructing haplotypes (Figure S1).

#### The overlapping sliding-window approach:

The sliding window approach constructs haplotypes with a fixed window length, *i.e.*, the number of adjacent SNPs (Huang *et al.* 2007). If the window is moved with a certain step size which is smaller than the window length adjacent windows are overlapping. In this study we chose the window length to be three, which is consistent with the length of our functional haplotypes, and the step size to be one.

#### The linkage disequilibrium approach:

The linkage disequilibrium (LD) approach groups SNPs into a haplotype if the LD between every two adjacent SNPs is equal or greater than a certain threshold, which allows physically close and non-randomly associated SNPs to be grouped together in the same haplotype (Barrett *et al.* 2005). In our study, the r^2^ statistic was used to measure LD (Hill and Robertson 1968) and the threshold was set to 0.9. Note, that in this method the constructed haplotypes may have different lengths. In all cases in which SNPs were not grouped into any haplotype, the single SNPs were considered as haplotypes of length one.

GWAS based on haplotypes constructed via the above two methods are referred as SWH GWAS and LDH GWAS respectively. The significance of haplotypes was also tested using the linear mixed model (Equation 1).

### Data sets

The study was based on published data of *Arabidopsis thaliana* from the 1001 Genomes Consortium (1001 Genomes Consortium 2016). The genotypic data contained 1,134 accessions with 11,458,975 single-nucleotide polymorphisms (SNPs). The phenotypic data that were considered was flowering time for plants grown at two different temperatures (10° and 16°), which included phenotypic values for 1,163 and 1,123 accessions respectively. Combining the genotypic and phenotypic data, 1,003 (970) accessions were used in the 10° (16°) data set. In the following the two data sets were referred as data set FT10 (10°) and FT16 (16°) respectively. Only bi-allelic SNPs were considered for the analyses. After removing the SNPs with missing rate above or equaling 0.1, the remaining missing values were imputed with IMPUTE2 (Howie *et al.* 2009; Howie *et al.* 2012). Linkage phases were determined by SHAPEIT (Delaneau *et al.* 2011). SNPs with minor allele frequency (MAF) below 0.05 were also removed. For subsequent analyses the resulting 756,005 and 754,656 SNPs were used for data set FT10 and FT16, respectively.

### Comparing FH GWAS with other methods using empirical data

We compared the performance of FH GWAS with that of SWH GWAS, LDH GWAS and the single SNP-based approach with the *Arabidopsis thaliana* data sets described in the previous section. The genome-wide thresholds for the different approaches were generally determined as *P* < 0.05 after Bonferroni correction for multiple testing (Dunn 1961). Thus for the SNP, SWH and LDH GWAS approaches, the thresholds were *P* < 0.05/m, where m is the number of SNPs or haplotypes constructed in total. The proportion of phenotypic variance explained by each of the significant SNPs or haplotypes was calculated as the adjusted *R^2^* in a linear regression model with intercept and the testing variable. For regions in which significant associations were detected in GWAS, annotated genes were retrieved from Araport11 (Cheng *et al.* 2017).

#### Implementation of FH GWAS:

In the procedure of preselecting SNPs, we filtered the markers with the threshold *P* < 0.01. Then we set the window size for searching haplotypes to be 50 kb and the number of SNPs in each haplotype to be three. Thus the linear mixed model (1) was performed for any triplet of candidate SNPs within 50 kb, testing the additive effects of each SNP, the epistatic effects for each pair of SNPs and the three-way epistatic effects. If at least two of the additive effects were significant with *P* < 0.05 and at least two of the pairwise epistatic effects were significant with *P* < 0.1, the triplet of SNPs was grouped into a functional haplotype. In the test of all functional haplotypes, we again applied the Bonferroni correction for multiple testing. But the threshold for FH GWAS needed further adjustment to account for the pre-testing procedure for single SNP effects and epistatic effects. So a more stringent threshold was determined as *P* < 0.05/(m+c), where m is the number of functional haplotypes and c is the number of tests performed in the pre-testing procedure. To select representative significant functional haplotypes, we used the stepwise linear regression model (Draper and Smith 2014) and applied a bidirectional elimination procedure minimizing the Schwarz Bayesian Criterion (Schwarz 1978).

### GWAS considering markers in perfect LD

SNPs in perfect LD ($r^{2}=1$) are virtually identical in GWAS models in the sense that they have the same estimated effects and P values. Thus for each group of SNPs in perfect LD, we recorded their positions and performed only one test in GWAS. This approach was termed SNP_LD_ GWAS. Let n_LD_ be the number of SNPs adjusted for perfect LD, meaning that SNPs in perfect LD were counted only once. Then the threshold for SNP_LD_ GWAS was *P* < 0.05/n_LD_.

For any two haplotypes consisting of three SNPs, they may share k SNPs (k = 0, 1, 2). If the remaining 3-k pairs of SNPs are in perfect LD respectively, the two haplotypes can be treated as identical in GWAS for the same reason as above. Thus our FH GWAS approach can also be adjusted by considering SNPs in perfect LD, which was termed FH_LD_ GWAS. Let m_LD_ be the adjusted number of functional haplotypes and c_LD_ be the adjusted number of tests performed in the pre-testing procedure. Then the new threshold for FH_LD_ GWAS was determined as *P* < 0.05/(m_LD_+c_LD_).

### Decay of linkage disequilibrium

The genome-wide decay of LD in the population of data set FT10 was estimated by a non-linear regression model using Hill and Weir’s function (Hill and Weir 1988). The same method was used to estimate the decay of LD for the five candidate regions detected in GWAS.

### Simulation study

Phenotypic data were simulated based on genotypic data of the 1,003 *Arabidopsis thaliana* accessions described previously (1001 Genomes Consortium 2016). Considering computational load, the simulations were restricted to all bi-allelic SNPs mapping to chromosome 2 regardless of MAF, in total 279,038 SNPs. In the simulation procedure three SNPs were always selected within a 50 kb window and main and epistatic effects were assigned to them. To clarify the influence of LD and MAF on the performance of haplotype-based GWAS, three ranges of LD (0-0.2, 0.3-0.6 and 0.7-1) between each pair of selected SNPs, three ranges of MAF (0-0.1, 0.2-0.3 and 0.4-0.5) for the selected SNPs and combinations thereof were considered. For each of the resulting nine simulation scenarios, the main effect of each SNP, the epistatic effects between each pair of SNPs and the three-term interaction effect were set to account for 6%, 3% and 1% of the explained proportion of genetic variance, respectively and the heritability was set to be 0.8. All remaining SNPs were required to contribute equally to the remaining proportion of explained genetic variance to simulate genetic background. Based on the resulting simulated phenotypic data, association mapping was performed (model 1) using the three SNPs of a particular haplotype individually and the haplotype. For each scenario, simulations were repeated 1,000 times.

### Data availability

All data analyzed in this study have been published previously (1001 Genome Consortium 2016). Phenotypic data were downloaded from AraPheno (https://arapheno.1001genomes.org/phenotypes/?sort=study&page=1). Genomic data were downloaded from the 1001 Genomes data center (http://1001genomes.org/data/GMI-MPI/releases/v3.1/, the data ‘1001genomes_snp-short-indel_only_ACGTN.v*cf*.gz’ was used in this study). FH GWAS was implemented using R (R Core Team 2017). The source code and sample data sets which are subsets of the original data set for running the code can be found at https://github.com/Fangv1/Functional_haplotype_GWAS/tree/master/. Supplemental files are available at figshare. Supplemental material available at figshare: https://doi.org/10.25387/g3.8967986.

## Results

### FH GWAS outperformed SNP-based and two other haplotype-based GWAS approaches

**Figure 2:**
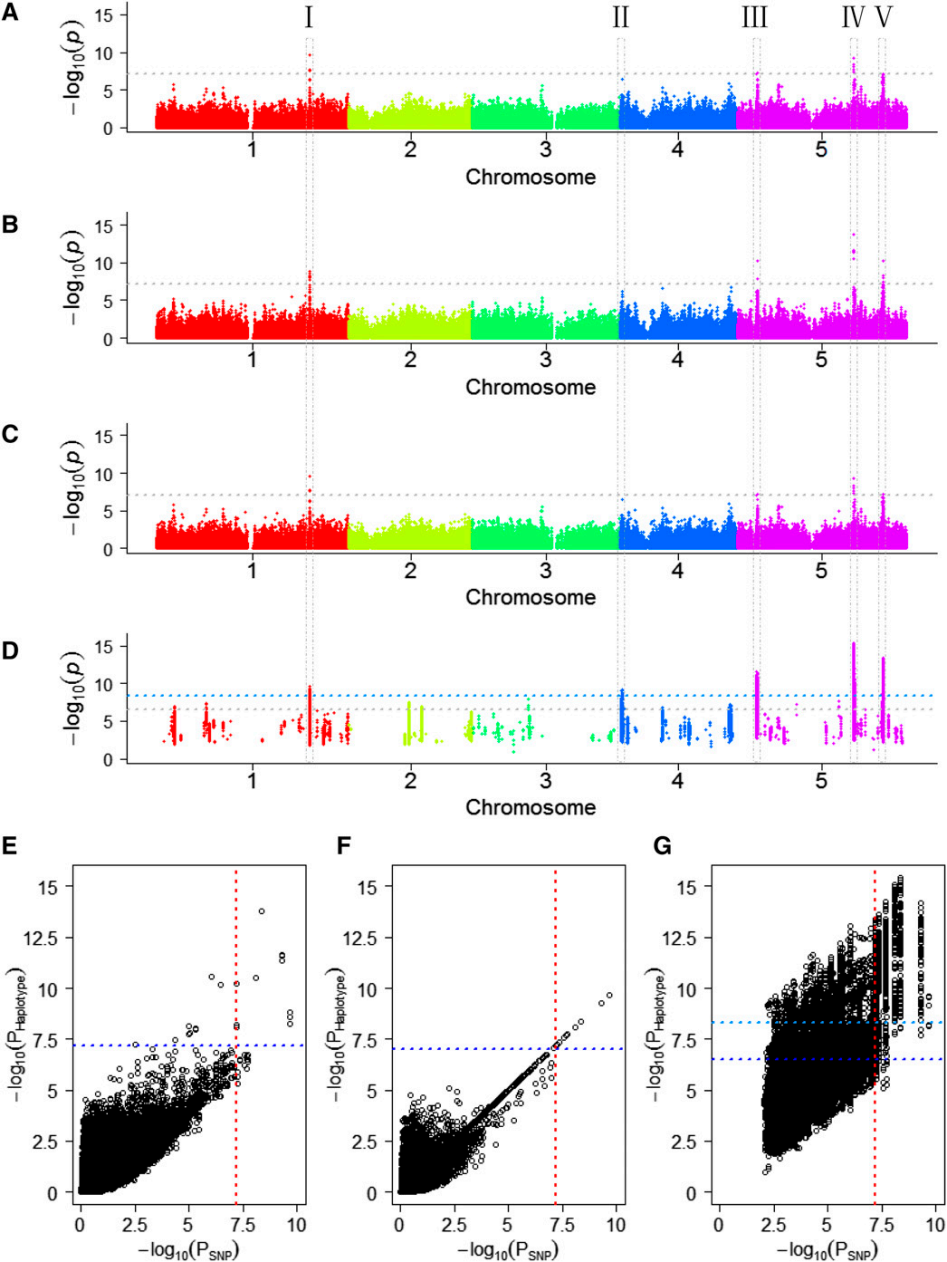
Association mapping results using four different GWAS approaches. (A-D) Manhattan plots illustrate the results for a single SNP-based (A) and three haplotype-based GWAS approaches for data set FT10 (B-D). Positions of SNPs or haplotypes on the five chromosomes are shown on the x axis relative to their -log_10_(*P*) values on the y axis. Haplotypes were constructed based on overlapping sliding-windows (B), linkage disequilibrium (C) or by using the functional haplotype approach (D). Thresholds after Bonferroni correction for multiple testing (Dunn 1961) (*P* < 0.05) are displayed as horizontal dotted gray lines. Taking into account the pre-testing procedure for single SNP main and epistatic effects implemented in the functional haplotype approach a more stringent threshold resulted that is indicated as a pale blue dotted line in panels (D) and (G). (E-G) Plots showing the -log_10_(*P*) values of haplotypes on the y axis relative to the -log_10_(*P*) values established by SNP-based GWAS for the most significant SNP of a corresponding haplotype on the x axis. The P value relationships for SWH, LDH and FH based GWAS are illustrated in panels (E), (F) and (G), respectively. Thresholds after Bonferroni correction for multiple testing (Dunn 1961) (*P* < 0.05) are indicated as horizontal dotted dark blue lines for haplotypes and vertical dotted red lines for single SNPs. The five regions in which significant associations were found were denoted with I to V and are marked by stippled lines.

In this study, data for flowering time in *Arabidopsis thaliana* accessions that had been cultivated at 10° and 16° were analyzed (1001 Genomes Consortium 2016), these compilations are referred to as data sets FT10 and FT16, respectively. Data set FT10 encompassed 1,003 accessions and after quality control 756,005 biallelic SNPs remained for subsequent analyses. To assess the performance of the proposed FH GWAS, we compared its results to those of single SNP-based GWAS and two other haplotype-based approaches in which haplotypes were either constructed using sliding-windows (SWH GWAS) or by considering LD of consecutive SNPs (LDH GWAS). The number of SNPs grouped into haplotypes and number of haplotypes analyzed in GWAS varied between the three approaches (Table S1). Applying Bonferroni correction for multiple testing (Dunn 1961) (*P* < 0.05) resulted in significance thresholds of -log_10_*P* = 7.03, -log_10_*P* = 7.18 and -log_10_*P* = 6.50 for LDH, SWH and FH GWAS, respectively. But for FH GWAS it was necessary to apply a further correction to account for the pre-testing procedure for single SNP effects and epistatic effects which preceded the construction of functional haplotypes (see **Materials and Methods** for details). Implementing this correction resulted in a more stringent threshold of -log_10_*P* = 8.29. Applying the different GWAS approaches, significant associations were found in five chromosome regions (Figure 2A-2D). Importantly, all regions that were identified by SNP-based, LDH and/or SWH GWAS were also found by FH GWAS. Four regions, I, III, IV and V, were identified by all methods, but region II on chromosome 4 solely showed significant association with flowering time using FH GWAS. For each of the significant functional haplotypes detected in region II, an additional association test was performed with only the main effects of the three SNPs in the haplotype. We found that in all cases the –log(P) values decreased by three to five orders of magnitude. This clearly showed the important contribution of epistatic effects to the overall effect of a functional haplotype.

For each haplotype the P value of the SNP for which the lowest P value had been observed in single SNP-based GWAS was compared to the one of the corresponding haplotype. The proportion of haplotypes showing significant associations that contained at least one SNP, which had passed the significance threshold in SNP-based GWAS, varied between the three different haplotype-based GWAS approaches (Figure 2E-2G). The highest proportion was found with 90.91% for LDH GWAS and the lowest one with 29.33% for FH GWAS (Table 1).

**Table 1:** Summary of significant associations in different genome-wide association studies obtained for the trait flowering time for *Arabidopsis thaliana* accessions using data set FT10

	SNP GWAS	SWH GWAS		LDH GWAS		FH GWAS	
Number of significant assocations	SNP	H_a_	H_b_	H_a_	H_b_	H_a_	H_b_
I (Chr1)	4	7	4	3	0	71	61
II (Chr4)	0	0	0	0	0	15	15
III (Chr5)	1	2	1	2	1	701	565
IV (Chr5)	5	6	1	5	0	19030	13900
V (Chr5)	1	5	2	1	0	4952	2963
Total	11	20	8	11	1	24769	17504

H_a_ refers to all significant haplotypes.

H_b_ represents significant haplotypes which did not contain any significant SNP.

### Accounting for linkage disequilibrium in functional haplotype-based GWAS

Strikingly, FH GWAS identified several thousand significant haplotypes whereas SNP-based GWAS and the other two haplotype-based GWAS approaches revealed few significant associations (Table 1, Figure 2). Inspection of the significant functional haplotypes in a given chromosome region revealed many subsets sharing one or two SNPs. For example, nine of the 15 significant haplotypes in region II had two SNPs in common. Moreover, the SNPs distinguishing these nine significant haplotypes were in high LD to each other (Figure S2). This exemplifies that many different significant functional haplotypes may result in cases in which significant haplotypes are made up of SNPs which are in high LD with other SNPs in the region. Taking into account which SNPs are in perfect LD to each other it is possible to restrict the FH GWAS analysis to those haplotypes which provide non-redundant information regarding additive and epistatic effects, called FH_LD_ GWAS hereafter. In data set FT10, the number of SNP combinations to be tested could be reduced in this manner from 8,932,265 to 2,460,993. Instead of 157,526 functional haplotypes in FH GWAS only 44,759 resulted in FH_LD_ GWAS. However, owing to a less stringent threshold of -log_10_*P* = 7.79 for FH_LD_ GWAS compared to -log_10_*P* = 8.29 for FH GWAS the number of significant associations increased (Table S2). Regardless whether FH GWAS or FH_LD_ GWAS were used multiple significant haplotypes were found in regions I to V. In addition, a single haplotype passed the significance threshold in FH_LD_ GWAS on chromosome 3 (Table S2, Figure 2, Figure S3).

### Representative significant haplotypes narrowed down the candidate regions

**Figure 3:**
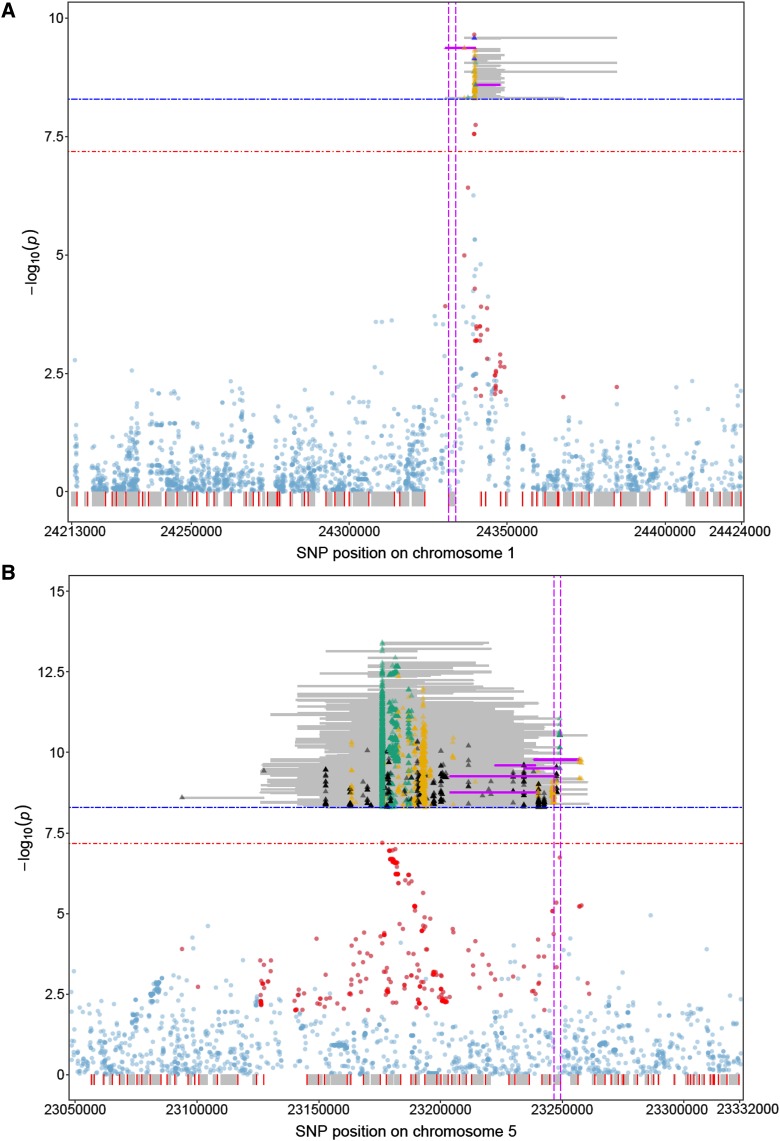
Details of significant associations for the trait flowering time revealed by SNP-based and functional haplotype-based GWAS in two chromosome regions. Panels (A) and (B) refer to the analysis of data set FT10 for regions I and V, respectively. SNP positions on the different chromosomes are shown on the x axis relative to the corresponding -log_10_(*P*) values on the y axis. The depicted regions reflect the chromosome segments for which overlapping functional haplotypes had been obtained, but only those functional haplotypes are shown which passed the stringent adjusted significance threshold of -log_10_(*P*) = 8.29 as gray or pink lines. Pink lines highlight representative significant functional haplotypes. The positions of the first and third SNP of a particular haplotype on the chromosome mark the beginning and end of the line, respectively. A colored triangle indicates the SNP of a haplotype for which the lowest P value was observed by SNP-based GWAS. P values ranging from 1 × 10^−4^ to 1 × 10^−2^, 1 × 10^−6^ to 1 × 10^−4^, 1 × 10^−8^ to 1 × 10^−6^ are represented as black, orange and green triangles, respectively. Blue triangles represent P values smaller than 1 × 10^−8^. The translucent pale blue and red dots correspond to the P values of SNPs obtained in single SNP-based GWAS, red dots represent those SNPs that were part of significant functional haplotypes. Below the x axis the coding regions of genes in the region are shown as gray boxes, 5′-regions are indicated as red lines. Two vertical pink dashed lines are used to mark the position of the coding region of the candidate gene. The red and blue horizontal stippled lines correspond to the significance thresholds for single SNP-based and FH GWAS, respectively.

**Figure 4:**
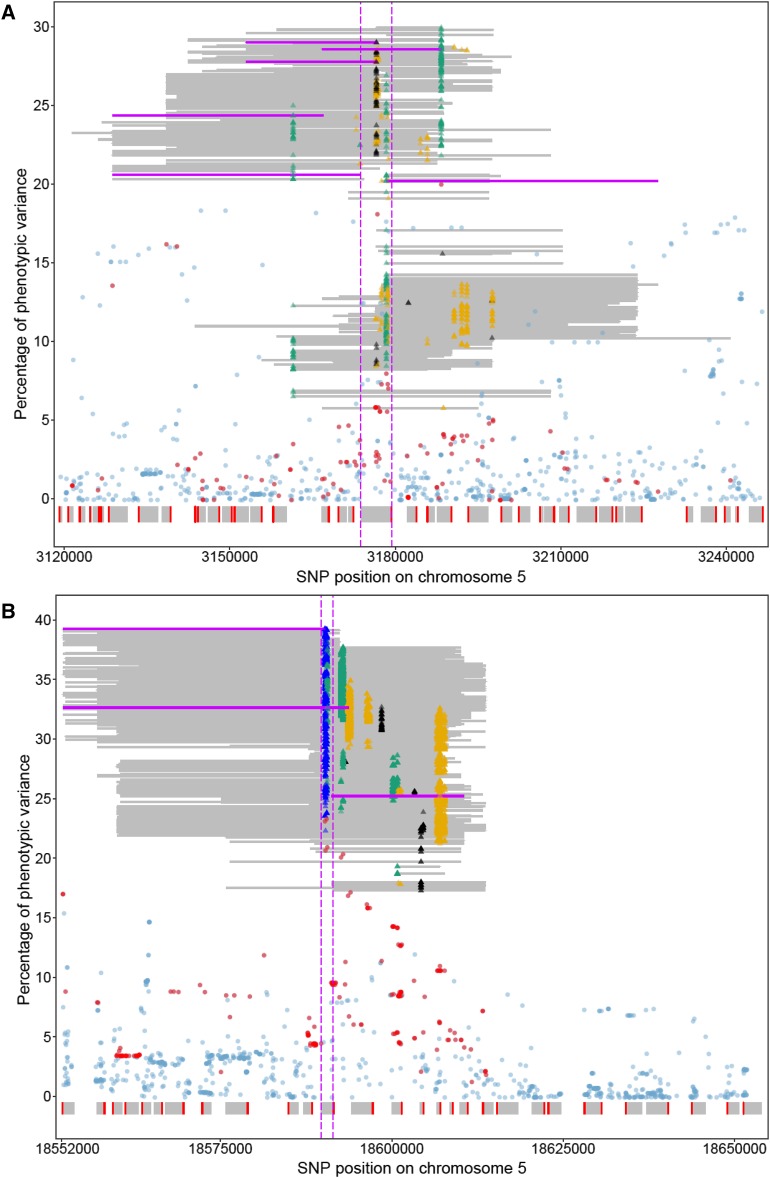
Proportions of explained phenotypic variance for the trait flowering time obtained by SNP-based and functional haplotype based GWAS in two different chromosome regions. Details for regions III and IV are illustrated for data set FT10 in panels (A) and (B), respectively. SNP positions on chromosome 5 and percentages of adjusted R^2^ values are shown on the x and y axes, respectively. Chromosome segments are illustrated for which overlapping functional haplotypes had been obtained, but only significant functional haplotypes are displayed as gray or pink lines. Representative significant functional haplotypes are indicated by pink lines. The beginning and end of the individual lines represent the chromosome positions of the first and third SNP of a particular haplotype, respectively. The SNP for which the lowest P value of a given significant functional haplotype was obtained is indicated as a colored triangle. Black, orange and green triangles represent P values ranging from 1 × 10^−4^ to 1 × 10^−2^, 1 × 10^−6^ to 1 × 10^−4^, 1 × 10^−8^ to 1 × 10^−6^, respectively. Blue triangles mark P values smaller than 1 × 10^−8^. Percentages of R^2^ determined for SNPs are displayed as translucent pale blue or red dots, those SNPs that were part of significant functional haplotypes are depicted in red. The coding regions of genes are shown as gray boxes and red lines represent 5′-regions. The position of the coding region of the candidate gene is marked by two vertical pink dashed lines.

Depending on the region, the mean size of the significant functional haplotypes, defined as the distance in base pairs between the outermost SNPs of a particular haplotype, varied from 10.6 to 41.3 kb in FH GWAS. Moreover, the size of chromosome segments in which overlapping significant functional haplotypes were found differed, ranging from 54.3 to 167.2 kb (Table S3). The sizes of the significant functional haplotypes in conjunction with their high number hampered the search for candidate genes. Variable selection methods were therefore used to reduce the number of significant functional haplotypes (see Materials and Methods for details). For data set FT10, two to six and two to eight representative haplotypes were selected per region in FH GWAS and FH_LD_ GWAS, respectively (Table S2). Taking into account the overlaps between all representative significant functional haplotypes of a given region and/or the area between them, small regions with few genes were detected (Figure 3, Figure 4, Figure S4, Table S4). In all cases a candidate gene was identified among these genes for which a role in flowering time control had been documented previously. *FT* (Kardailsky *et al.* 1999; Corbesier *et al.* 2007) represents a candidate gene for region I on chromosome 1 (Figure 3A). *DOG1* (Huo *et al.* 2016) and *FLC* (Michaels and Amasino 1999; Li *et al.* 2014) are part of regions III and IV on chromosome 5, respectively (Figure 4). In these three areas, the significant SNPs that were significantly associated with the trait flowering time were also located in the candidate gene itself or in its immediate vicinity. It is important to note that the proportions of phenotypic variance explained by the representative significant haplotypes were in four out of five analyzed regions higher than those determined for any of the SNPs in these chromosome segments (Figure 4, Table S5). In region V on chromosome 5, the only SNP significantly associated with the trait flowering time mapped approximately 60 kb apart from the region with the candidate gene *VIN3* (Sung and Amasino 2004) which was indicated by the representative significant functional haplotypes in FH GWAS (Figure 3B). In region II that had only been detected using FH GWAS and FH_LD_ GWAS, *CCT*/*CRP*/*MED12* (Imura *et al.* 2012) was identified as candidate gene (Figure S2, Figure S4).

### FH GWAS for the trait flowering time at two different growth temperatures

Association studies in which the trait flowering time had been comparatively analyzed for accessions cultivated at 10° and 16° had revealed fewer significant SNP associations in the latter data set (1001 Genomes Consortium 2016). It was therefore of interest to extend the performance comparisons of SNP-based and FH GWAS to data set FT16, in which phenotypic data for 970 accessions and 754,655 biallelic SNPs that had passed quality control had been compiled. Two regions showing significant associations were identified by SNP-based GWAS as well as FH GWAS using data set FT16 (Figure S5), these corresponded to regions III and IV that had also been found for plants cultivated at 10°. In contrast, region II on chromosome 4 was solely identified by FH GWAS, regardless which of the two data sets was analyzed (Figure 2, Figure S5). Significant associations in regions I and V were not found in data set FT16. However, for FT16, FH GWAS detected in three additional regions located on chromosomes 1, 2 and 3 between one and three significant haplotypes associated with the trait flowering time (Figure S5).

The three regions, which were identified by FH GWAS in both data sets, were analyzed in more detail. A comparison of the results revealed that the chromosome segments in which the representative significant functional haplotypes were found showed large overlaps in both data sets (Table S4) implying that the same three candidate genes underlie the trait flowering time in these regions (Figure S6). Interestingly, such congruence was not observed if for example the candidate SNPs that were considered for the construction of functional haplotypes were compared in the two data sets. Although around 650 candidate SNPs were identified in each of the two data sets, only 390 were in common. Similar results were found by assessing the SNPs that were grouped into haplotypes (Table S6). Although 67,865 and 57,479 functional haplotypes had been considered in GWAS using the two data sets, only 3,606 were in common between both data sets. A similar trend was seen if only those haplotypes were considered that had passed the GWAS significance thresholds. None of the representative significant functional haplotypes were identical in the two data sets (Table S6).

### The influence of linkage disequilibrium and minor allele frequencies on the power of functional haplotype-based GWAS

**Figure 5:**
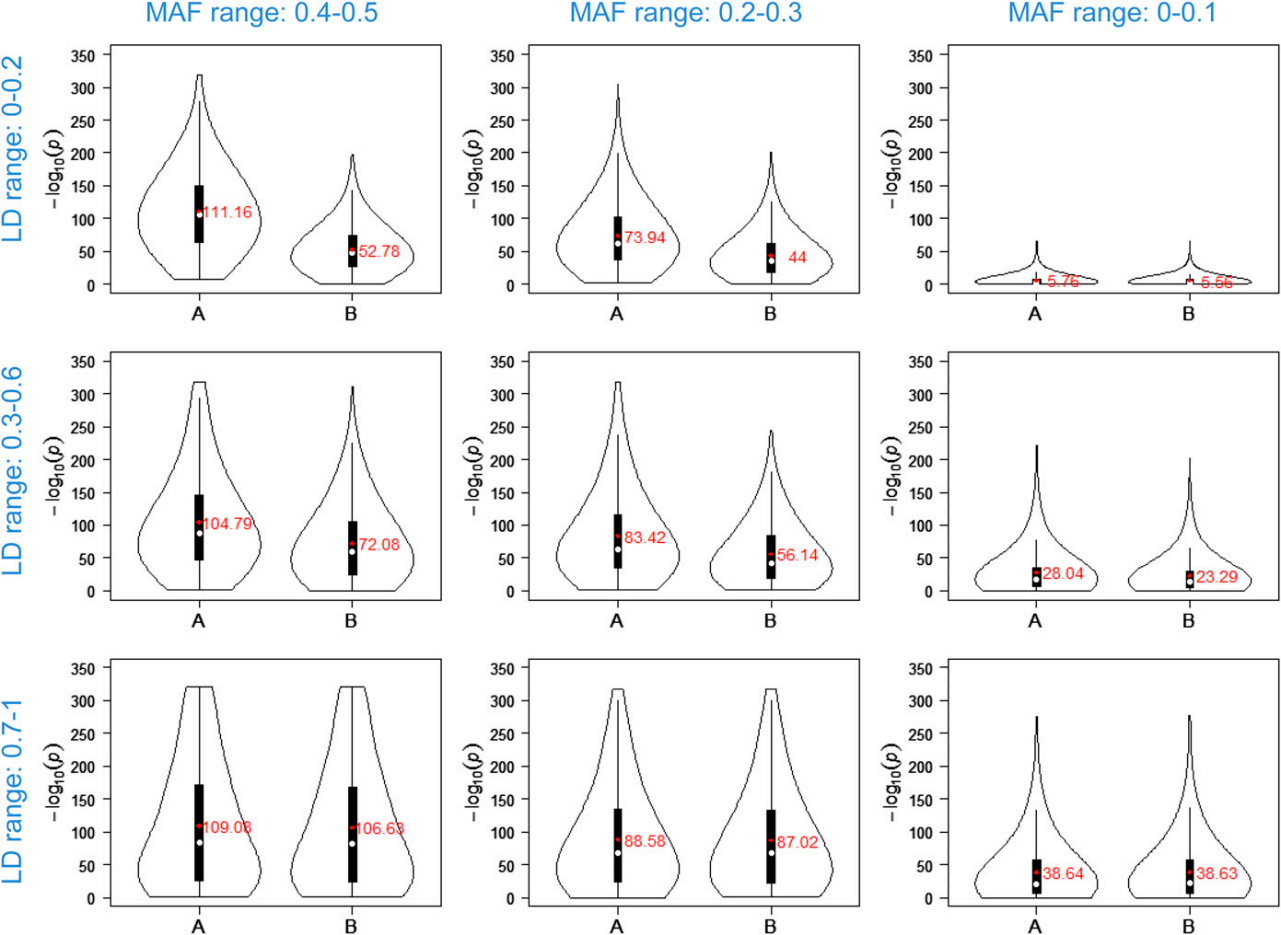
Comparison of P value distributions for FH GWAS and SNP-based GWAS obtained for nine different simulation scenarios. The violin plots show the distributions of P values after 1,000 simulation runs. Plots are arranged in order of decreasing MAF and increasing LD range. The y axis corresponds to the -log_10_(P values). ‘A’ represents the P values of haplotypes and ‘B’ the P values of the most significant SNPs of the particular haplotypes. The black vertical line corresponds to the 95% confidence interval and the black vertical box represents the interquartile range. The white and red dots mark the median and mean values, respectively. The latter values are indicated in the plots.

Simulation studies were performed to gain insight under which circumstances FH GWAS outperforms SNP-based GWAS. Specifically, it was analyzed how the minor allele frequency (MAF) of the SNPs making up a particular haplotype and the LD between them influenced the results of FH GWAS, therefore three MAF and LD ranges each as well as all of their combinations were considered (see **Materials and Methods** for details). The P value distributions obtained for the haplotypes using the nine different simulation scenarios are shown in Figure 5 side-by-side with the results for the most significant SNPs of the different haplotypes. Mean P values were inversely correlated with the MAF range in FH GWAS and SNP-based GWAS, regardless which LD range was analyzed. In scenarios in which the MAF range was kept constant, inverse correlations were seen between the mean P values and the LD range. Exceptionally, analysis of the highest MAF range revealed very similar mean P values in case of FH GWAS for the three different LD ranges. In four out of the nine scenarios tested, the mean P values obtained for FH GWAS clearly outperformed those of SNP-based GWAS, in each of these four scenarios more than 96% of the haplotypes revealed lower P values compared to the values that had been established by SNP-based GWAS for the most significant SNPs of these haplotypes (Table S7). This was not the case in the five scenarios in which the lowest MAF range and/or the highest LD range were analyzed. The same trends were observed regarding the proportion of phenotypic variance explained by the haplotypes and the most significant SNPs of the different haplotypes (Figure S7).

## Discussion

We devised a haplotype-based GWAS approach, FH GWAS, for studying complex quantitative traits which capitalizes on a novel way in which main and epistatic effects among markers are considered to group SNPs into haplotypes. In FH GWAS we first select SNPs with a mild threshold for main effects and then search for combinations of consecutive and/or non-consecutive SNPs in a genomic region of defined size requiring certain significance for epistatic effects. In this way, only those SNPs having true contribution to the haplotype effects via additive and/or epistatic effects are combined into functional haplotypes. Thus, FH GWAS is able to overcome the constraints of combining redundant SNPs in high LD into haplotypes and meanwhile it avoids exhausted search for optimal combinations of SNPs which is too time-consuming. It is therefore expected to be more powerful than SNP-based and other haplotype-based GWAS approaches, which was confirmed by the empirical analyses for the trait flowering time in *Arabidopsis thaliana* using the data from the 1001 Genomes Consortium (1001 Genomes Consortium 2016). Our FH GWAS approach detected not only all regions, which were detected in the SNP-based and the other two haplotype-based approaches, but also a new candidate region on chromosome 4 for plants cultivated at 10° and 16° (Figure 2, Figure S5). The FH GWAS approach can be generally applied to any quantitative trait in any homozygous species for which populations with appropriate SNP coverage and of suitable size are available. If multiple traits are studied, the functional haplotypes have to be constructed for each trait separately as the tests of marker main and epistatic effects are trait-dependent. Thus, FH GWAS enhances the power of GWAS in a way that is tailored for each trait, however, it has a higher computational load than other haplotype-based GWAS approaches in which solely consecutive SNPs are considered for haplotype construction.

### On the implementation of functional haplotype-based GWAS

The first step of FH GWAS is a mild preselection of SNPs according to their main effects in order to reduce the computational load for the remaining steps. Thus, it is necessary for high density SNP data sets generated for example by whole genome sequencing projects as used in this study (1001 Genomes Consortium 2016). Theoretically, the significance of a haplotype effect can be solely a result of significant epistatic effects, or cumulative (non-)significant main and epistatic effects among the SNPs. The preselection of SNPs is therefore dispensable and can be omitted if the computational load is acceptable.

In the second step of the procedure, the construction of functional haplotypes, there are two important parameters to be determined, namely the size of the window in which the functional haplotypes are constructed and the number of SNPs to be grouped into haplotypes. The window size is essentially determined by the extent of LD in the population, however, gene density should also be considered. A too small window size leads to high LD among markers within the window, reducing the advantage of haplotypes according to the results of the simulation study (Figure 5), whereas a too large window size may yield functional haplotypes that span large regions on the chromosome involving many candidate genes. For the *Arabidopsis thaliana* population considered in this study, the window size was set to be 50 kb, where the LD (measured as r^2^) decayed to 0.03 (Figure S8A). On average 22 genes mapped to intervals of this size in the *Arabidopsis thaliana* Col-0 genome (Arabidopsis Genome Initiative 2000). Interestingly, in the region with the steepest LD decay (Figure S8B), region I, median and mean haplotype sizes were substantially smaller than in the other four regions (Table S3).

The number of SNPs in each haplotype is directly relevant to the power of association test, which decreases as the number of haplotype alleles increases. Usually only a small number of SNPs can be afforded unless the population size is very large, because the number of haplotype alleles grows exponentially with an increasing number of SNPs constituting the haplotype. It is also limited by the computational load because allowing more SNPs in a haplotype results in more possible combinations of SNPs to be tested. Thus in this study the number of SNPs in each haplotype block was set to be three.

### Functional haplotypes boosted power of GWAS by exploiting statistical epistasis

The construction of functional haplotypes rests upon interaction effects among markers, which was termed statistical epistasis in quantitative genetics (Moore and Williams 2005). In general, the estimation of statistical epistasis is not directly relevant to biological mechanisms of gene interactions (Carlborg and Haley 2004), although some simulation studies showed that various functional dependency patterns of genes could result in significant statistical epistasis (Gjuvsland *et al.* 2007). As we observed many significant functional haplotypes consisting of SNPs with non-significant main effects even in the region where SNPs with strong main effect were detected (Figure 3B), a variable-selection algorithm was applied to select representative haplotypes. This step was of crucial importance to narrow down the regions, which needed to be inspected for the presence of candidate genes. In each candidate region, detailed analyses of the representative haplotypes revealed several distinct two- or three-locus genotype-phenotype patterns. Moreover, although three of the candidate genes were identified in two different data sets, none of the representative significant functional haplotypes were identical in these two data sets (Table S5). These findings made it unlikely that the statistical epistasis exploited by the significant haplotypes reflected a biological mechanism of gene interactions but also revealed that the cumulative statistical epistatic effects among SNPs in haplotypes indeed enhanced the power of FH-GWAS. Hence, the approach is useful for detecting new candidate regions, which cannot be detected using SNP-based or other haplotype-based GWAS approaches. Previously, haplotype-based methods were used to boost power in GWAS mainly for incomplete genotype data (McCarthy *et al.* 2008), whereas our study showed that FH GWAS is a promising method even if almost complete genotype information is available such as whole-genome sequencing data.

### Further development of functional haplotype-based GWAS

In this study, FH GWAS was applied to an *Arabidopsis thaliana* population consisting of pure homozygous lines. Hence, the haplotype phase was known and only the additive-by-additive epistasis was considered in the construction of functional haplotypes. A generalization of the FH GWAS method for heterozygous populations is possible as algorithms inferring haplotype phases (Browning and Browning 2011) can be applied if the haplotype phase is unknown. It may, however, be necessary to consider other types of epistasis, additive-by-dominance and dominance-by-dominance, when constructing functional haplotypes. Note, that in these cases the relationship between haplotype effects and marker epistatic effects was only illustrated in two- or three-locus examples but not formally proved in general case (Conti and Gauderman 2004; Schaid 2004; Jiang *et al.* 2018). Thus, further theoretical and empirical studies are needed to develop an optimal strategy of FH GWAS for heterozygous populations.
